# Potential Novel Role of Membrane-Associated Carbonic Anhydrases in the Kidney

**DOI:** 10.3390/ijms24044251

**Published:** 2023-02-20

**Authors:** Seong-Ki Lee, Walter F. Boron, Rossana Occhipinti

**Affiliations:** 1 Department of Physiology and Biophysics, School of Medicine, Case Western Reserve University, Cleveland, OH 44106, USA; 2 Department of Medicine, School of Medicine, Case Western Reserve University, Cleveland, OH 44106, USA; 3 Department of Biochemistry, School of Medicine, Case Western Reserve University, Cleveland, OH 44106, USA

**Keywords:** transporters, carbonate, bicarbonate, acid–base homeostasis, cell membranes, renal tubules

## Abstract

Carbonic anhydrases (CAs), because they catalyze the interconversion of carbon dioxide (CO_2_) and water into bicarbonate (HCO_3_^−^) and protons (H^+^), thereby influencing pH, are near the core of virtually all physiological processes in the body. In the kidneys, soluble and membrane-associated CAs and their synergy with acid–base transporters play important roles in urinary acid secretion, the largest component of which is the reabsorption of HCO_3_^−^ in specific nephron segments. Among these transporters are the Na^+^-coupled HCO_3_^−^ transporters (NCBTs) and the Cl^−^-HCO_3_^−^ exchangers (AEs)—members of the “solute-linked carrier” 4 (SLC4) family. All of these transporters have traditionally been regarded as “HCO_3_^−^“ transporters. However, recently our group has demonstrated that two of the NCBTs carry CO_3_^2−^ rather than HCO_3_^−^ and has hypothesized that all NCBTs follow suit. In this review, we examine current knowledge on the role of CAs and “HCO_3_^−^” transporters of the SLC4 family in renal acid–base physiology and discuss how our recent findings impact renal acid secretion, including HCO_3_^−^ reabsorption. Traditionally, investigators have associated CAs with producing or consuming solutes (CO_2_, HCO_3_^−^, and H^+^) and thus ensuring their efficient transport across cell membranes. In the case of CO_3_^2−^ transport by NCBTs, however, we hypothesize that the role of membrane-associated CAs is not the appreciable production or consumption of substrates but the minimization of pH changes in nanodomains near the membrane.

## 1. Introduction

Carbonic anhydrases (CAs) are ubiquitous metalloenzymes that play a fundamental role in numerous vital processes, including cellular metabolism, carbon dioxide (CO_2_) and ion transport, and acid–base homeostasis [1,2,3,4,5]. These enzymes are important because they permit rapid interconversion of CO_2_ and bicarbonate (HCO_3_^−^), thereby promoting the rapid buffering of acid or alkali loads and facilitating—to various degrees—the diffusion of CO_2_, HCO_3_^−^, H^+^, and other buffers [5]. For example, in red blood cells (RBCs), intracellular CA provides a mechanism for the efficient carriage of CO_2_ from peripheral tissues to the lungs [5]. In the kidneys, as we shall see later, the synergistic action of soluble and membrane-associated CA activity permits efficient reabsorption of filtered HCO_3_^−^.

CAs are also linked to a variety of pathological processes, including renal tubular acidosis, osteopetrosis, osteoporosis, and tumor proliferation [6,7,8,9,10,11,12]. Inhibition of CAs has important pharmacological implications, for example, in the treatment of glaucoma, epilepsy, acute mountain sickness, and, more recently, even cancer [13,14,15,16,17]. CAs may also be useful as markers for cancer diagnosis [17,18,19].

Because of their involvement in physiological and pathological processes, these enzymes continue to be the focus of current investigations. CA was discovered in 1933 in bovine RBCs by Meldrum and Roughton during their study of the interactions between CO_2_ and HCO_3_^−^ in blood [20]. The first three isozymes to be identified were CA A (later renamed CA III), CA B (later CA I), and CA C (later CA II) [21,22,23,24]. We now recognize eight evolutionally unrelated families of CAs: α-, β-, γ-, δ-, ζ-, η-, θ-, and ι-CAs [2,13,25,26,27]. α-CAs, which include CAs I–III, are found in numerous eukaryotic and prokaryotic organisms and are the most widely studied and characterized because of their importance in higher animals [28,29]. β-CAs are found in both eukaryotes (e.g., algae, plant chloroplast, and fungi) and prokaryotes. Recently, β-CAs have also been identified in animals, particularly in invertebrates [28,29,30,31]. γ-CAs are mainly found in archaea and some bacteria, and δ- and ζ-CAs are expressed in some marine diatoms [28,29,32]. The η-CAs are found in the protozoan *Plasmodium* species, which cause malaria in humans [33]. In the past few years, investigators have identified θ- [34] and ι-CAs [25] in marine diatoms.

While most CAs have a zinc ion (Zn^2+^) in their active site, the γ- and ζ- families may contain different metals ions (e.g., iron [35] or cobalt [36] in γ-CAs and cadmium [37] in ζ-CAs). Some ι-CAs may not require any metal ion in their active site [27,38].

**Figure 1:**
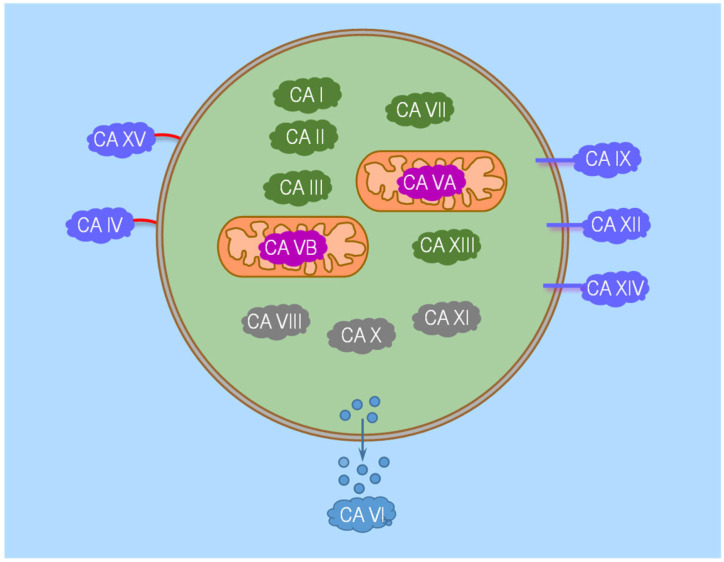
Cartoon illustrating the sub-cellular localization of the currently known α-CA family members. According to their sub-cellular localization, α-CAs can be classified into four groups: cytosolic (CA I, CA II, CA III, CA VII, CA VIII, CA X, CA XI, and CA XIII), mitochondrial (CA VA and CA VB), secretory (CA VI), and membrane-associated (CA IV, CA IX, CA XII, CA XIV, and CA XV). The cytosolic isozymes are all active (indicated in dark green) except for CA VIII, X, and XI (in grey), which are also called CA-related proteins (CARPs). Of the membrane-associated CAs, CA IV and CA XV are glycosylphosphatidylinositol (GPI)-linked, whereas CA IX, CA XII, and CA XIV are transmembrane. Note that the mitochondrial CA VA and CA VB are encoded by two different genes [43,44].

In this review, we focus exclusively on the α-family, which predominates among animals. The α-CA family comprises thirteen enzymatically active isozymes and three inactive ones. Figure 1 shows their different sub-cellular localizations (i.e., cytosolic, mitochondrial, secretory, and membrane-associated). Each member of the α-CA family has a distinct combination of kinetic properties and tissue-specific distribution. For example, CA II and CA III are both found in the cytosol but have the highest and lowest catalytic activities, respectively [39,40,41]. CA II is widely expressed throughout the body, whereas CA III is expressed mainly in muscle cells [2]. Moreover, members of the α-CA family have different susceptibilities to inhibitors. For example, CA II can be easily blocked by acetazolamide (ACZ) and topimirate (i.e., very small amounts of inhibitor can block enzyme activity). CA IV can be blocked more easily by ACZ than topimirate [42].

In the following sections, we examine the role of CA isozymes in renal acid–base physiology by reviewing established knowledge as well as new insights obtained by recent work from our group on the substrate identity of “HCO_3_^−^” transporters of the “solute-linked carrier 4” (SLC4) family. After a brief overview of the members of the SLC4 family that play a role in renal acid–base physiology, we systematically review CA expression, sub-cellular localization, and functional interaction with SLC4 family proteins along the nephron segments that engage in acid secretion, including HCO_3_^−^ reabsorption. We conclude by suggesting that a role of membrane-associated CAs may be to reduce local pH changes caused by the transmembrane movement of carbonate (CO_3_^2−^) via the Na^+^-coupled HCO_3_^−^ transporter (NCBT) subfamily of SLC4 rather than to produce the substrate(s) for acid–base transport by NCBTs, as traditionally thought.

## 2. Catalytic Mechanism of α-Carbonic Anhydrases

In the absence of CAs, the interconversion of CO_2_ and HCO_3_^−^ is a two-step bidirectional process:

(1) $${\mathrm{CO}}_{2}+H_{2}O\;⇌\;H_{2}{\mathrm{CO}}_{3}\;⇌\;{{\mathrm{HCO}}_{3}}^{-}+H^{+}.$$

In the first of the two sequential reactions (each governed by its own pK), CO_2_ hydration describes the combination of CO_2_ with water (H_2_O) to form carbonic acid (H_2_CO_3_). Dehydration describes the opposite direction. In the second reaction, H_2_CO_3_ dissociates into HCO_3_^−^ and H^+^; this reaction, too, is reversible.

The two-step interconversion of CO_2_ and HCO_3_^−^ is extremely slow because of the high activation energy of the first of the two reactions. A CA enzyme (activator) lowers the overall activation energy by bypassing the H_2_CO_3_ intermediate as the CA catalyzes the thermodynamically equivalent reaction:

(2) $${\mathrm{CO}}_{2}+H_{2}O\;⇌\;{{\mathrm{HCO}}_{3}}^{-}+H^{+}.$$

**Figure 2:**
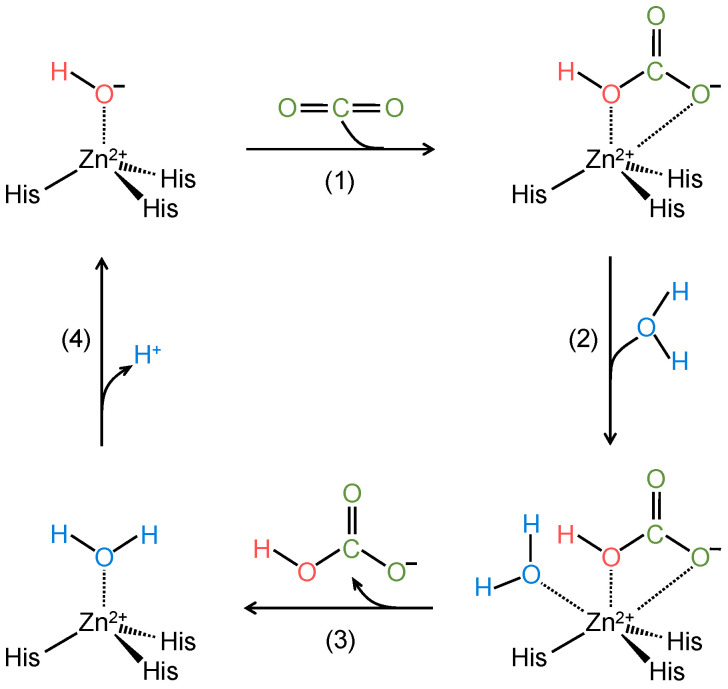
Schematics of the catalytic mechanism of action of α-CAs. In step #1, CO_2_ (green-colored) reacts with a zinc-bound hydroxyl (OH^−^, red-colored), generating a coordinated HCO_3_^−^. In step #2, H_2_O (blue-colored) binds to zinc (Zn^2+^). In step #3, HCO_3_^−^ dissociates from Zn^2+^, thereby leading to the catalytically inactive form of the enzyme. In step #4, release of a H^+^ ion from the Zn^2+^-bound H_2_O molecule regenerates the Zn^2+^-bound OH^−^ group, thereby restoring the catalytic active form of the enzyme. His = histidine residue.

Figure 2 shows the catalytic mechanism of reaction (2) for all α-CAs. Known as the zinc-hydroxide mechanism [45,46], the central catalytic step—when viewed in the forward direction in reaction (2)—is the reaction of CO_2_ with a zinc-hydroxide (i.e., Zn^2+^-OH^−^). The presence of the hydroxyl group rather than H_2_O (i.e., Zn^2+^-H_2_O, a less strong nucleophile than Zn^2+^-OH^−^) is necessary for the enzyme to be active and able to start the forward cycle [47].

In the state illustrated in the upper left of Figure 2, the Zn^2+^ of the α-CAs coordinates with three conserved histidine (His) residues and one hydroxyl group. Because in biological enzymes, Zn^2+^ can have up to six coordination sites, when α-CAs are in the Zn^2+^-OH^−^ state, the Zn^2+^ has two free coordination sites [48,49]. In step #1 of the forward reaction cycle, the nucleophile O^−^ (red-colored) in the hydroxyl group performs a nucleophilic attack on the carbon of CO_2_ (green-colored), generating HCO_3_^−^ on the active site. An electron-rich O^−^ (green-colored) on the HCO_3_^−^ molecule makes a fifth coordination with Zn^2+^. In step #2, H_2_O interacts with Zn^2+^ via its O atom (blue-colored). This sixth coordination to Zn^2+^ destabilizes the two bonds between Zn^2+^ and HCO_3_^−^ so that, in step #3, HCO_3_^−^ dissociates from the enzyme active site. In step #4, Zn^2+^ acts as a Lewis acid, promoting deprotonation of the coordinated H_2_O, thereby releasing H^+^ (blue-colored) and regenerating the Zn^2+^-OH^−^ (we now morph the color from blue to red to restart the cycle) form of the enzyme [50].

The presence of different amino acids—with different positions and orientations—near the active site provides different catalytic activity and kinetics to the various CA isozymes. For example, the presence of histidine at position 64 (His-64) of CA II favors H_2_O deprotonation (i.e., step #4 in Figure 2) by transferring H^+^ to the buffer in the media. Indeed, site-directed mutagenesis studies have shown that replacing this “shuttle histidine” with either alanine or glutamine inhibits CA II catalytic activity [51,52]. In the case of CA III—which has a structure very similar to that of CA II but much lower catalytic activity—substitution of the naturally occurring lysine-64 (in CA III) with His-64 enhances its enzymatic activity [39]. In addition, the presence of bulky amino acids next to His-64 in CA II, or equivalently next to His-88 in CA IV, can also decrease catalytic activity. Other residues can modulate catalytic activity. Among them, threonine (Thr) 199 in CA II is important for stabilizing HCO_3_^−^ in the active site by forming a network of hydrogen bonds [53,54].

The α-CA family members are all enzymatically active except for the three cytosolic forms CA VIII, X, and XI that, for lack of one or more of the three conserved histidine residues in the active site, are unable to perform the cycle illustrated in Figure 2. These three CAs are known as CA-related proteins (CARPs). Despite being apparently linked to various diseases, their physiological role is not well understood [55]. An intriguing possibility is that, like the CA-like domain of the receptor protein tyrosine phosphatase γ (see below), the CARPs may be molecular sensors of CO_2_ and/or HCO_3_^−^.

## 3. Carbonic Anhydrases in Nanodomains Adjacent to the Cell Membrane

In addition to reactions (1) and (2) above, the chemistry of HCO_3_^−^ also includes the dissociation of this species into carbonate (CO_3_^2−^) and H^+^:

(3) $${{\mathrm{HCO}}_{3}}^{-}\;⇌\;{{\mathrm{CO}}_{3}}^{2-}+H^{+}.$$

In contrast to reaction (2), reaction (3) is very fast and does not rely on the action of a catalyst. Another difference is their respective pK values, ~6.1 at 37 °C for reaction (2) and ~10.3 for reaction (3). As a consequence, reaction (2) is dominant at physiological pH, and the majority of CO_2_-related carbon species in the body is in the form of HCO_3_^−^.

It is informative to examine how the transmembrane movements of CO_2_, HCO_3_^−^, H^+^, and CO_3_^2−^ as well as how membrane-associated CAs (all of which are on the outer surface of the plasma membrane; see Figure 1) affect the chemical equilibria of reactions (2) and (3).

**Figure 3:**
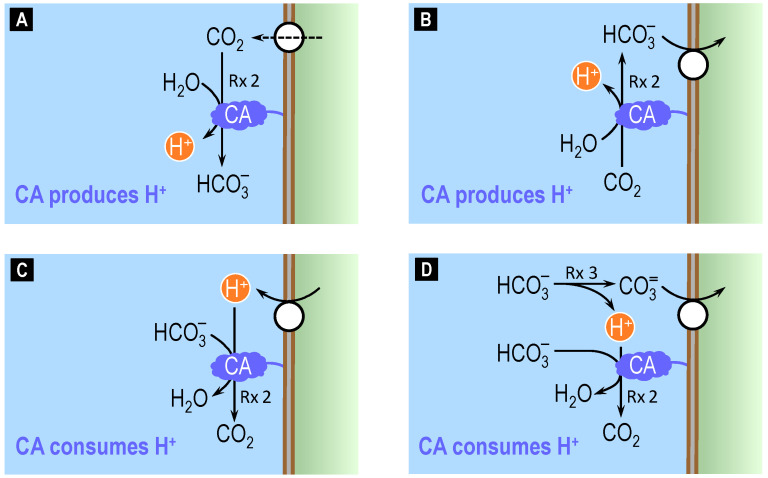
Interaction of fluxes of CO_2_, HCO_3_^−^, H^+^, and CO_3_^2−^ with CA-catalyzed reactions on the outer surface of a cell. (**A**) If CO_2_ exits the cell, CA tends to minimize the rise in [CO_2_] at the outer surface (oS) of the cell membrane, and maximize the fall in pH_oS_. (**B**) If HCO_3_^−^ enters the cell, CA tends to minimize the fall in [HCO_3_^−^]_oS_ and maximize the fall in pH_oS_. (**C**) If H^+^ exits the cell, CA tends to minimize the rise in [H^+^]_oS_ (i.e., minimize the fall in pH_oS_). (**D**) If CO_3_^2−^ enters the cell, CA tends to minimize the fall in [CO_3_^2−^]_oS_ (indirectly) and the fall in pH_oS_ (directly). Rx = reaction; the number following the abbreviation ‘Rx’ indicates the numbering of the reaction in the main text. CA = carbonic anhydrase.

When CO_2_, HCO_3_^−^, H^+^, or CO_3_^2−^ move across a cell membrane, they perturb the chemical equilibria of reactions (2) and (3) in the nanodomain near the outer surface (oS) of the membrane, as illustrated in Figure 3. Comparable, though opposite, reactions occur at the inner surface of the membrane (not shown). In panels A–D, we orient all fluxes in the direction that would produce a fall in pH_oS_. If CO_2_ moves out of the cell (Figure 3A), CA tends to reestablish the chemical equilibrium of reaction (2) near the outer surface by consuming the exiting CO_2_ (i.e., minimizing the rise in [CO_2_]_oS_) to produce HCO_3_^−^ and H^+^ (i.e., accentuating the rise in [H^+^]_oS_/fall in pH_oS_). If CO_2_ were entering the cell, the CA would do just the opposite. Thus, in both situations (i.e., independently of the direction of CO_2_ movement), CA tends to minimize changes in [CO_2_]_oS_ but maximize changes in pH_oS_.

If HCO_3_^−^ moves into the cell (Figure 3B), CA tends to produce HCO_3_^−^ (i.e., minimizing the fall in [HCO_3_^−^]_oS_) as well as H^+^ (i.e., accentuating the rise in [H^+^]_oS_/fall in pH_oS_). If HCO_3_^−^ were exiting the cell, the CA would do just the opposite. Thus, independently of the direction of HCO_3_^−^ movement, the CA tends to minimize changes in [HCO_3_^−^]_oS_ but maximize changes in pH_oS_.

If H^+^ moves out of the cell (Figure 3C), CA tends to consume H^+^ (i.e., minimizing the rise in [H^+^]_oS_) as well as HCO_3_^−^ (i.e., accentuating the fall in [HCO_3_^−^]_oS_). If H^+^ were entering the cell, the CA would do just the opposite. Thus, independently of the direction of H^+^ movement, the CA tends to minimize changes in pH_oS_.

A more complex scenario occurs when CO_3_^2−^ moves across the cell membrane because CO_3_^2−^ movement first produces a major perturbance in the chemical equilibrium of reaction (3) near the outer surface of the membrane, followed by a large secondary effect on the equilibrium of reaction (2) because [HCO_3_^−^]_oS_ >> [CO_3_^2−^]_oS_ under physiological conditions. If CO_3_^2−^ enters the cell (Figure 3D), reaction (3) tends to replenish the lost CO_3_^2−^, thereby producing H^+^ near the outer surface of the membrane. Catalyzing reaction (2), CA will then consume much of the newly formed H^+^. If CO_3_^2−^ were exiting the cell, the CA would do just the opposite. Thus, independently of the direction of CO_3_^2−^ movement, the CA tends to minimize not only changes in [CO_3_^2−^]_oS_ but also changes in pH_oS_.

In summary, all four panels in Figure 3 show us that CA near the membrane tends to stabilize the concentration of the transported solute, regardless of direction. Based on intuition, one might predict that these actions, in principle, would universally accelerate transport. However, this is not true. In the case of CO_2_ (Figure 3A), the work from our group [56,57,58]—which extends the earlier work of Gutknecht and colleagues [59]—shows that the stimulation of CO_2_ transport by CAs is quite large. The reason is that the CA-catalyzed reaction can have very large effects on [CO_2_]_oS_ because pH_oS_ >> pK, so that [HCO_3_^−^]_oS_ >> [CO_2_]_oS_. In the case of HCO_3_^−^ (Figure 3B), CA has negligible effects on the transport rate, as described below. We would expect as much: because [HCO_3_^−^]_oS_ >> [CO_2_]_oS_ under physiological conditions, the CA-catalyzed reaction has little impact on [HCO_3_^−^]_oS_. In the case of H^+^ (Figure 3C), the effect of CA on transport rate has, to our knowledge, not been tested. However, we suspect that the impact of CA might be muted, again because the high [HCO_3_^−^]_oS_/[CO_2_]_oS_ ratio would tend to reduce the extent of the forward reaction in Figure 3C. Finally, in the case of CO_3_^2−^ (Figure 3D), CA has a negligible impact on the transport rate, as we discuss below. We would expect as much: because [HCO_3_^−^]_oS_ >>>> [CO_3_^2−^]_oS_ under physiological conditions, the rate of the reaction HCO_3_^−^ → CO_3_^2−^ + H^+^ is apparently little influenced by CA-dependent changes in [H^+^]_oS_.

Continuing the summary, the upper row of Figure 3 shows that, for fluxes of CO_2_ (Figure 3A) or HCO_3_^−^ (Figure 3B), CA magnifies pH changes in the nanodomain near the membrane. Conversely, the lower row of Figure 3 shows that, for fluxes of H^+^ (Figure 3C) or CO_3_^2−^ (Figure 3D), CA minimizes pH changes in the nanodomain near the membrane.

Below, we show that it is possible to exploit the chemistry of reactions (2) and (3), as well as a variety of biophysical approaches, to identify the substrates carried by “HCO_3_^−^” transporters.

## 4. Carbonic Anhydrases and the Identification of Substrates of SLC4 Family Members

In theory, “HCO_3_^−^” transporters could carry any of the solutes involved in reactions (2) and (3). Distinguishing among them has been challenging because, in contrast with non-labile ions (e.g., sodium, potassium), the solutes of reactions (2) and (3) are interchangeable (i.e., they can be converted into each other), making direct measurements virtually impossible. Some investigators have resorted to surrogate substrates or kinetic approaches [60,61,62,63,64,65,66]. However, neither approaches are definitive: (i) No surrogate can mimic the real physicochemical properties of the substrates under consideration [67]. (ii) The only definitive conclusion that can come from kinetic approaches—and even then, only under favorable circumstances—is to rule out false hypotheses. Although kinetic studies can support a model, they can never rule one out. Thus, although various investigators may have had their hypotheses based on surrogate or kinetic data, none of these conclusions—by definition—could have been definitive.

An advancement towards solving this technical conundrum was the theoretical observation that the combination of CA inhibitors and their opposite effects on pH changes could help distinguish HCO_3_^−^ vs. CO_3_^2−^ transport across cell membranes [68,69,70]. However, early studies with CA inhibitors and pH measurements could not determine unambiguously the identity of the transported substrate because of limitations in the experimental system (native tissue), which almost certainly comprised an unknown mixture of acid–base transporters. Moreover, in these earlier studies, the investigators did not consider the possibility that “HCO_3_^−^” transporters could carry H^+^ or CO_2_. Interestingly, preliminary work from our group suggests that the electrogenic Na/HCO_3_ cotransporter-1 (variant A) can conduct CO_2_ [71,72].

In the following sections, after a brief overview of the members of the SLC4 family of “HCO_3_^−^” transporters, we review a recent study from our group in which we were able to identify unambiguously the nature of the substrates carried by the “HCO_3_^−^” transporters of the SLC4 family.

### 4.1. Brief Overview of the SLC4 Family Members

Mammalian “HCO_3_^−^” transporters belong to two major gene families, namely SLC4 and SLC26. Nine of the ten SLC4 members carry “HCO_3_^−^”. To date, some members of the SLC26 family appear to carry “HCO_3_^−^” [73,74], an example of which is SLC26A4 or pendrin [75,76]. In addition, HCO_3_^−^ can cross membranes via Cl^−^ channels, such as the cystic fibrosis transmembrane conductance regulator (CFTR) and γ-aminobutyric acid (GABA) receptor channel [77].

Here, we focus on the members of the SLC4 family that carry “HCO_3_^−^” because of their predominant role in renal HCO_3_^−^ reabsorption.

The mammalian SLC4 family includes ten genes (*SLC4A1-5*; *SLC4A7-11*) that encode a group of ten functionally diverse integral membrane proteins. All but *SLC4A11* encode proteins that transport HCO_3_^−^ or a HCO_3_^−^-related species, such as CO_3_^2−^—we refer to all of these substrates as “HCO_3_^−^”. These transporters can be either Na^+^-independent or Na^+^-dependent. The three Na^+^-independent members are the anion exchangers (AE1-3; products of *SLC4A1-3* genes), which carry HCO_3_^−^ in exchange for Cl^−^. The five Na^+^-dependent members are the Na^+^-coupled bicarbonate transporters (NCBTs), which carry Na^+^ and “HCO_3_^−^” in the same direction. The NCBTs include the two electrogenic Na^+^/HCO_3_^−^ cotransporters NBCe1 (*SLC4A4*) and NBCe2 (*SLC4A5*) that carry electrical current, the two electroneutral Na^+^/HCO_3_^−^ cotransporters NBCn1 (*SLC4A7*) and NBCn2 (*SLC4A10*), and the electroneutral Na^+^-driven Cl^−^/HCO_3_^−^ exchanger NDCBE (*SLC4A8*). The remaining ninth “HCO_3_^−^” transporter is the protein encoded by *SLC4A9*, currently named AE4. However, despite being called AE4, it is still controversial whether this protein is a Na^+^-independent Cl^−^/HCO_3_^−^ exchanger [78,79,80] or a Na^+^-dependent HCO_3_^−^ transporter [81,82]. A tenth member of the SLC4 family is the bicarbonate transporter-related protein-1 (BTR1, *SLC4A11*), which is no longer believed to be a HCO_3_^−^ transporter but rather a H^+^ (or OH^−^) conducting protein [83,84].

Members of the SLC4 family are expressed throughout the body and are essential for regulating intracellular and whole-body pH, and for transporting acid–base equivalents across many epithelia. These proteins are implicated in a variety of diseases. For example, mutations of AE1 have been associated with type I distal renal tubular acidosis (RTA) and hereditary spherocytosis [85,86,87]. Mutations of NBCe1 have also been linked to type II proximal RTA, glaucoma, migraine, and suicidal ideation [88,89,90].

Many reviews on the SLC4 family are available, and we refer the interested reader to these for more details (refs. [91,92,93]).

### 4.2. Theoretical Role of Carbonic Anhydrase in Distinguishing Bicarbonate versus Carbonate versus Proton Transport across Cell Membranes

**Figure 4:**
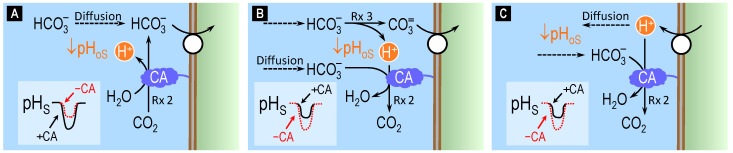
Theory of bicarbonate influx vs. carbonate influx vs. proton efflux across cell membranes. (**A**) The entry of HCO_3_^−^ into the cell leads to the production of H^+^ (i.e., a decrease in pH_oS_; inset, lower left corner: solid black trace) via the CA-catalyzed reaction CO_2_ + H_2_O → HCO_3_^−^ + H^+^ (indicated as ‘Rx 2’) at the outer surface (oS) of the cell membrane. Blocking CA (i.e., applying a CA inhibitor) reduces H^+^ production (i.e., the decrease in pH_oS_; dashed red trace). (**B**) The entry of CO_3_^2−^ into the cell leads to the production of H^+^ (i.e., a decrease in pH_oS_; solid black trace) via the reaction HCO_3_^−^ → CO_3_^2−^ + H^+^ (indicated as ‘Rx 3’) at the outer surface of the cell membrane. Blocking CA (i.e., applying a CA inhibitor) reduces H^+^ consumption by ‘Rx 2’ (i.e., increases the fall in pH_oS_; dashed red trace). (**C**) The exit of H^+^ from the cell leads to an immediate increase in [H^+^]_oS_ (i.e., a decrease in pH_oS_; solid black trace) which is mitigated by the CA-catalyzed reaction HCO_3_^−^ + H^+^ → CO_2_ + H_2_O at the outer surface of the cell membrane. Blocking CA (i.e., applying a CA inhibitor) reduces H^+^ consumption by ‘Rx 2’ (i.e., increases the fall in pH_os_; dashed red trace). In model (**C**), the effect of blocking CA is the same as in model (**B**). Rx = reaction; the number following the abbreviation ‘Rx’ indicates the numbering of the reaction in the main text. CA = carbonic anhydrase.

Figure 4 illustrates the three possible models of acid–base transport that we explored in our study [73]: HCO_3_^−^ influx (panel A), CO_3_^2−^ influx (panel B), and CO_2_/HCO_3_^−^-stimulated H^+^ efflux (panel C). For simplicity, we consider only the case in which a base enters the cell. Similar conclusions can be reached for the case in which a base exits the cell. We also omit the accompanying movements of Na^+^ and/or Cl^−^ and the corresponding postulated transporter stoichiometry of the members of the SLC4 that we studied.

If the transporter mediates HCO_3_^−^ entry into the cell (Figure 4A), the result will be a decrease in [HCO_3_^−^]_oS_. The lost HCO_3_^−^ can be replenished by either diffusion (indicated by the dashed arrow), which does not affect pH_oS_ or reaction (2). Because reaction (2) produces H^+^, it will cause a decrease in pH_oS_ (see inset, lower left corner of panel A: solid black trace). Blocking CA (i.e., applying a CA inhibitor) will reduce H^+^ production, thereby causing a smaller decrease in pH_oS_ (dashed red trace).

If the transporter mediates CO_3_^2−^ entry into the cell (Figure 4B), the result will be a decrease in [CO_3_^2−^]_oS_ that triggers reaction (3), thereby replenishing CO_3_^2−^ but also producing H^+^. The consequence is a decrease in pH_oS_ (inset: solid black trace). Consuming some of this newly formed H^+^ will be reaction (2) catalyzed by CA. Therefore, blocking CA will reduce H^+^ consumption via reaction (2), causing a further decrease in pH_oS_ (dashed red trace). Thus, the blockade of CA in model A vs. model B has opposite effects on pH_oS_.

If the transporter, stimulated by CO_2_ or HCO_3_^−^, mediates H^+^ efflux from the cell (Figure 4C), the result will be an immediate increase in [H^+^]_oS_, as reflected by a fall in pH_os_ (inset: solid black trace). This triggers both H^+^ diffusion away from the membrane and the CA-catalyzed reaction (2), both of which mitigate the rise in [H^+^]_oS_. Blocking CA will slow H^+^ consumption, magnifying the decrease in pH_oS_ (dashed red trace).

Comparing the hypothetical solid black vs. the dashed red traces in the insets of Figure 4A–C, we see that the effect of CA blockade on the direction of the pH_oS_ change should, in principle, allows one to distinguish HCO_3_^−^ transport on the one hand (Figure 4A) from CO_3_^2−^ or H^+^ transport on the other (Figure 4B,C).

### 4.3. Surface pH Studies Supporting Carbonate as the Substrate of NCBTs

In order to test the three possible models of transport in Figure 4, we co-expressed in oocytes (i) NBCe1-A ± CA IV as a test case for electrogenic NCBTs; (ii) AE1 ± CA IV as a test case for electroneutral AEs and (iii) NDCBE ± CA IV as an additional test case for electroneutral NCBTs. In all of these three cases, we exploited (i) the chemistry of reactions (2) and (3) at the outer surface of the plasma membrane, (ii) the CA inhibitor acetazolamide (ACZ), (iii) measurements of pH_oS_, (iv) heterologous expression of the proteins in *Xenopus* oocytes, and (v) mathematical simulations.

NBCe1-A appears to mediate the isodirectional movement of 1 Na^+^ and 2 HCO_3_^−^ ions (i.e., 1:2 stoichiometry), [93,94], as we could represent by doubling all stoichiometry values in Figure 4A. In principle, NBCe1-A could also move 1 Na^+^ and 1 CO_3_^2−^ in the same direction, as indicated by Figure 4B, or 1 Na^+^ and 2 H^+^ in the opposite direction in a CO_2_ or HCO_3_^−^ stimulated process, as we could represent by doubling all stoichiometry in Figure 4C. All three models are thermodynamically equivalent.

Because NBCe1-A is electrogenic, we simultaneously measured changes in NBC current (*I*_NBC_) and pH_oS_ with microelectrodes by shifting membrane voltage (*V*_m_) using two-electrode voltage clamping technique. Details on this technical approach can be found in ref. [95]. Our data show that inhibition of CA IV by ACZ amplifies pH_oS_ changes, thereby allowing us to rule out HCO_3_^−^ transport (i.e., Figure 4A).

In order to distinguish between the transport of 1 Na^+^ and 1 CO_3_^2−^ in the same direction vs. 1 Na^+^ and 2 H^+^ in opposite directions, we introduced a powerful new set of tools: we analyzed the amount of H^+^ that appears at the cell surface (measured as a change in pH_oS_) per electrical charge carried (*e*^−^, measured as change in *I*_NBC_) in each model. Using the well-characterized H^+^ channel H_V_1 to calibrate our experimental data and a reaction-diffusion mathematical model that simulates our experiments with oocytes, we were able to predict the amount of H^+^/*e*^−^ that appears at the cell surface. The mathematical model is an extension of the one employed in previous studies [56,57,58] and includes reactions (1) and (3) as well as the non-CO_2_/HCO_3_^−^ buffers (i.e., HEPES in the extracellular space to mimic the composition of our perfusion solution and the intrinsic cytosolic buffers). Comparing the model prediction with our experimental data, we were able to rule out both the Na^+^ + 2 HCO_3_^−^ model (simulation predicted very low H^+^/*e*^−^; see Figure 5A in Ref. [73]) and the Na^+^-2H^+^ exchange model (simulation predicted very high H^+^/*e*^−^; see Figure 5C in Ref. [73]). In fact, our data matched very closely the predictions for the transport of CO_3_^2−^ (simulation predicted moderate H^+^/*e*^−^; see Figure 5B in Ref. [73]). Thus, we definitively conclude that NBCe1-A cannot carry either HCO_3_^−^ or H^+^ and most likely carries 1 Na^+^ and 1 CO_3_^2−^ in the same direction.

In the cases of the electroneutral transporters AE1 and NDCBE, we could not use the H^+^/*e*^−^ approach. Instead, we acid loaded the cytoplasm of oocytes by exposure to CO_2_/HCO_3_^−^ (the CO_2_ enters the cell and generates HCO_3_^−^ + H^+^) and then used intracellular-pH microelectrodes to monitor the subsequent recovery (i.e., increase) of intracellular pH (pH_i_), which reflects the activity of the transporter. In addition, we monitored pH_oS_ changes during the rise in pH_i_.

Our data on AE1 show that inhibition of CA IV by ACZ reduces pH_oS_ changes, thereby allowing us to rule out the transport of CO_3_^2−^ (Figure 4B) or H^+^ (Figure 4C) and confirm what investigators had long believed; namely, AE1 transports HCO_3_^−^ (Figure 4A).

Our data on NDCBE show that inhibition of CA IV by ACZ amplifies pH_oS_ changes–the opposite of what we observed with AE1–thereby allowing us to rule out HCO_3_^−^ transport (Figure 4A).

In summary, our study shows that NBCe1-A almost certainly carries CO_3_^2−^ or a related substrate (e.g., the NaCO_3_^−^ ion pair: NaHCO_3_^−^ ⇌ Na^+^ + CO_3_^2−^), AE1 does indeed carry HCO_3_^−^, and NDCBE does not carry HCO_3_^−^. We suggest that similar studies will likely show that all AEs transport HCO_3_^−^ and all NCBTs transport CO_3_^2−^.

Finally, our approach can also be used for clarifying the nature of the substrates of transporters (e.g., SLC26 transporters) and channels that traditionally have been thought to mediate the movement of “HCO_3_^−^”.

## 5. Relative Abundance of Members of the SLC4 and α-CA Families in Human Kidney

Before systematically examining the members of the SLC4 and α-CA families involved in renal HCO_3_^−^ reabsorption, we used data from a recently published human study to obtain information on the relative abundance of mRNA and proteins in the kidney [96]. Although the transcriptome analysis is comprehensive and unbiased, the protein expression is relative to the tissue with the highest expression of that protein. Therefore, we cannot truly compare relative amounts of different proteins in the kidney. In some cases, mRNA and protein levels do not correlate well. The study’s authors suggest that this discrepancy could be due to the specificity and affinity of different antibodies directed to different proteins, as well as the non-linearity of immunohistochemistry assays, or to post-translational modifications of proteins (e.g., secretion and proteolysis). We note that static mRNA levels do not need to correlate with static protein levels, let alone with the disposition of the protein (e.g., cellular localization, posttranslational modification) that is physiologically relevant. Thus, even though these data represent an impressive amount of work and may provide important insights for further experiments, one must interpret such data with caution.

The results of this study [96] are included in the Human Protein Atlas database (see “http://www.proteinatlas.org”) (accessed on 20 January 2023).

### 5.1. SLC4 Family Members

Based on the RNAseq data of Fagerberg and coworkers [96], we classify the nine members of the SLC4 family that carry “HCO_3_^−^” into four major categories (very low, low, medium, and high), using the normalized tags per million (nTPM) value as an index of mRNA level (Table 1). For proteins, we simply use Fagerberg’s categories (as low, medium, and high) to describe expression levels [96]. It is noteworthy—but see caveats above regarding protein values—that *SLC4A4* (NBCe1) has both high mRNA and protein values. On the other hand, *SLC4A7* (NBCn1) and *SLC4A10* (NBCn2) both have very low or low mRNA levels but high protein values.

Below, we provide information on current knowledge of cellular and sub-cellular localization of members of this family along the nephron.

### 5.2. α-CA Family Members

For the 15 human α-CA family members (note: humans lack *CA15*), Table 2 summarizes the mRNA and protein expression levels following the same approach that we employed for the SLC4 family. We note that the *CA2* and *CA12* genes produce both the highest nTPM and protein values. On the other hand, *CA4* has medium mRNA but low protein levels.

These results are consistent with our current knowledge that CA II and CA XII are highly expressed in the kidneys but apparently are in conflict with the current impression that CA IV is similarly important. However, see the caveats above regarding reported protein levels. Note that CA IX overexpression has been associated with renal carcinoma [17].

Below, we provide information on current knowledge of cellular and sub-cellular localization of members of this family along the nephron.

## 6. Carbonic Anhydrases along the Nephron

The first report of CA activity in the kidneys dates back to 1941 when Davenport and Wilhelmi detected CA activity in the renal cortex of dogs, cats, and rats [98]. The identification of CA in the kidney was an important step toward understanding renal acid–base physiology. 

The role of CA in urinary acidification emerged when investigators observed that the administration of CA inhibitors reduces the titratable acidity of the urine. Höber was the first to report that the addition of sulfanilamide in the perfusion fluid blocks urinary acidification in frog kidneys [99]. Studies by Pitts and Alexander also supported the role of CA in urinary acidification. These authors demonstrated that the two theories prevailing at the time on the mechanism of urinary acidification—reabsorption of bicarbonate vs. reabsorption of alkaline phosphate—could account only for a relatively small fraction of the maximum titratable acidity of the urine. Thus, they suggested that secretion of H^+^ into the lumen was the only mechanism that could explain the amount of acid in the urine [100,101,102,103,104]. Pitts and Alexander correctly hypothesized that secretion of H^+^ in renal tubules occurs in exchange for some filtered luminal cation, most likely Na^+^ (as postulated earlier by Homer Smith [105,106]). Moreover, experiments with sulfonamide led these authors to suggest that the likely source of secreted H^+^ was intracellular CO_2_ hydration and that intracellular CA catalysis was an important part of this process [101]. During this time, the distal tubule was considered the main site of urinary acidification. Although some investigators had also suggested that the proximal tubule (PT) could play a role in urinary acidification [107,108,109], it was only in 1960 that Gottschalk and colleagues provided direct evidence on proximal acidification [110]. By performing microperfusion studies in rat kidneys, these authors observed that the fluid of the PT acidified to a pH of ~6.8, consistent with the hypothesis that most bicarbonate reabsorption (>85%) occurs in this segment.

Micropuncture and microperfusion studies exploiting CA inhibitors demonstrate that HCO_3_^−^ reabsorption in PTs (i) strongly depends on the presence of luminal/membrane-associated and cytosolic CA and (ii) occurs via H^+^ secretion into the lumen and not by direct absorption of HCO_3_^−^ across the apical membrane [111,112,113,114,115]. Burg and colleagues, working with isolated perfused tubules, demonstrated that the PT and thick ascending limb (TAL) are major nephron segments responsible for reabsorbing HCO_3_^−^ and that they both rely on CA and a Na-coupled H^+^-secretion mechanism [116,117,118,119]. McKinney and Burg confirmed that the collecting duct contributes to urinary acidification by reabsorption of bicarbonate in a CA-dependent manner [116]. However, contrary to what had been believed, they found that H^+^ secretion in this segment is independent of Na^+^ transport [116]. 

Our current knowledge of renal CA and its localization along the nephron is the result of numerous biochemical, immunocytochemical, and histochemical studies [24,120,121,122,123,124,125,126]. In 1975 Wistrand and coworkers, by employing affinity-chromatography techniques, were able to isolate CA from human kidneys and demonstrate that this CA was the same CA II of human RBCs [122]. These authors observed that a small percentage of the CA activity is not cytosolic but originates from a membrane-associated CA localized in both apical and basolateral membranes [127]. We now know that CA II, CA IV, and CA XII are the most prominent CA isozymes in the human kidney, with cytosolic CA II accounting for over 95% of the total renal CA activity and membrane-associated CA IV and CA XII accounting for the remaining 5% [24,111,120]. Although CA XIV is apparently not important in the human kidney, and the human genome lacks the *CA15* gene, both of these membrane-associated CAs play a role in the rodent kidney [2,120].

Below we describe the mechanisms of HCO_3_^−^ reabsorption along the nephron with special emphasis on our current knowledge about the localization and functional role of the three renal CA isozymes in humans. The same proteins or processes that mediate HCO_3_^−^ reabsorption also generate “new HCO_3_^−^”. The only difference is that, in the case of HCO_3_^−^ reabsorption, the H^+^ secreted into the lumen titrates HCO_3_^−^, whereas in the case of new-HCO_3_^−^ formation, the secreted H^+^ titrates NH_3_ (producing NH_4_^+^ secretion) or buffers like phosphate and creatinine (producing titratable acidity).

### 6.1. Proximal Tubule

PTs are responsible for reabsorbing ~80% to ~85% of filtered HCO_3_^−^. Most of this HCO_3_^−^ (after the luminal reaction HCO_3_^−^ + H^+^ → H_2_O + CO_2_) moves in the form of CO_2_ from the lumen, across the apical membrane (AM), and into the cytosol. After reconversion to HCO_3_^−^, the carbon crosses the basolateral membrane (BLM) in the form of CO_3_^2−^ or HCO_3_^−^.

**Figure 5:**
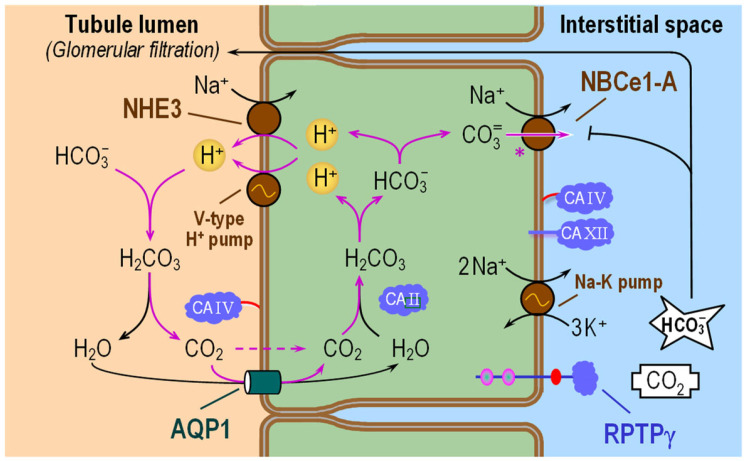
Cell model of HCO_3_^−^ reabsorption in renal proximal tubule. In the lumen, filtered HCO_3_^−^ and H^+^—secreted by NHE3 and V-type H^+^ pump—react to form CO_2_ and H_2_O under the catalytic action of CA IV. Apical entry of CO_2_ and H_2_O via AQP1 leads to the intracellular formation of HCO_3_^−^ and H^+^ under the catalytic action of CA II. NBCe1-A carries CO_3_^2−^(further ionized form of HCO_3_^−^) out of the cell across the basolateral membrane. The pink symbol ‘*’ near NBCe1-A denotes uncertainty about NBCe1-A stoichiometry. For details, see text. NHE3 = Na-H exchanger 3; V-type H^+^ pump = Vacuolar-type H^+^ pump (or V-ATPase); AQP1 = aquaporin 1; CA II, CA IV and CA XII = carbonic anhydrase II, IV and XII; NBCe1-A = electrogenic Na/HCO_3_ cotransporter (1 variant A); RPTPγ = receptor protein tyrosine phosphatase-γ.

Figure 5 illustrates the mechanisms of the preceding series of events. Mediating the majority of the H^+^ secretion at the AM is Na-H exchanger 3 (NHE3), which uses the inward Na^+^ gradient—established by the Na-K pump at the BLM—to exchange luminal Na^+^ for intracellular H^+^ [128,129,130]. Mediating a smaller fraction of H^+^ secretion is the apical vacuolar-type H^+^ pump (or V-type H^+^ pump), which uses ATP hydrolysis to energize the extrusion of H^+^ from the cell. A recent study on rat kidneys indicates that one variant of NBCn2 is present in the AM, where it could be responsible for the direct reabsorption of perhaps 20% of the reclaimed HCO_3_^−^ [131].

In the lumen, secreted H^+^ combines with filtered HCO_3_^−^, thereby producing CO_2_ and H_2_O under the catalytic action of CA IV. The newly formed CO_2_ and H_2_O cross the AM mostly through the water channel aquaporin AQP1 [132,133,134,135]. In the cytosol, CA II promotes rapid conversion of CO_2_ and H_2_O into HCO_3_^−^ and the first of two H^+^. This newly formed H^+^ then recycles back into the lumen, whereas the newly produced HCO_3_^−^ will further dissociate to form a second H^+^ plus CO_3_^2−^ and exit the cell across the BLM in the form of CO_3_^2−^ via NBCe1-A [136]. This second H^+^ also recycles back into the lumen. Because the debate continues as to whether, in PT cells, NBCe1-A truly operates with a 1Na^+^:3“HCO_3_^−^” stoichiometry or, as in most other cells, with a 1Na^+^:2“HCO_3_^−^” stoichiometry, in Figure 5 we show a single CO_3_^2−^ only (i.e., consistent with a 1:2 stoichiometry). An apparent 1:3 stoichiometry could be explained, for example, with the exit of an additional HCO_3_^−^ ion via NBCe1-A. Alternatively, the apparent necessity to invoke a 1:3 stoichiometry could be the result of measurement resolution—that is, using available macroscopic measurements of [Na^+^] and [“HCO_3_^−^”] near the inner and outer sides of the membrane rather than the relevant but unavailable measurements in the nanodomains adjacent to NBCe1-A.

Recent work is consistent with the notion that the receptor protein tyrosine phosphatase-γ (RPTPγ) at the BLM modulates H^+^ secretion/HCO_3_^−^ reabsorption by activating a signaling mechanism in response to changes in basolateral [CO_2_] and [HCO_3_^−^], [137,138,139,140].

#### 6.1.1. Subcellular Localizations of CA Isozymes

CA II is present in the cytosol, and the glycosylphosphatidylinositol (GPI)-linked CA IV is expressed on both AM and BLM (less on the BLM) of the S1 and S2 segments of the PT [120,141]. CA XII is expressed only on the BLM [9].

Regarding RPTPγ, previous studies from our group suggest that the extracellular CA-like domain (CALD) senses CO_2_ or HCO_3_^−^ and the phosphatase domain turns on a downstream signaling cascade that reaches key acid–base transporters [137,138,140]. Interestingly, the CALD of RPTPγ, compared to CA II, lacks several key residues necessary for catalysis, including two of the three histidine residues essential for coordinating Zn^2+^. If a physiological role of RPTPγ is indeed to sense basolateral CO_2_ and HCO_3_^−^, one would expect it to lack catalytic activity (i.e., the molecule should not interconvert the solutes that it is detecting), as is indeed the case [142,143].

#### 6.1.2. Functional Interactions with Acid–Base Transporters

As illustrated in Figure 5, the role of apical CA IV in HCO_3_^−^ reabsorption is to convert luminal HCO_3_^−^ to CO_2_ for uptake across the AM. The role of cytosolic CA II is to convert the CO_2_ entering across the AM to HCO_3_^−^ and H^+^. Thus, both CA IV and CA II enhance the gradient that favors CO_2_ uptake across the AM into the PT cell. CA II also provides cytosolic H^+^ for extrusion by NHE3 and V-type H^+^ pump across the AM, and HCO_3_^−^ (which generates CO_3_^2−^) for transport by NBCe1-A across the BLM. 

One group, following the lead of others working on AE1 in RBCs, reports that CA II binds to the C-terminus of NBCe1-A, creating a transport metabolon that enhances NBCe1-A activity [144,145]. In addition, others have reported that CA IV binds to the 4th extracellular loop of NBCe1-A, also stimulating NBCe1-A activity [146]. These papers are consistent with the idea that cytosolic CA II provides the substrate for NBCe1-A (HCO_3_^−^ or CO_3_^2−^), while CA IV at the outer surface of the BLM dissipates the substrate [146]. However, our group detected neither binding of CA II to NBCe1-A, nor acceleration of transport, even after fusing CA II to the C-terminus of NBCe1-A [147,148,149]. Moreover, our recent work (pH_S_ experiments of Figure 2 in ref. [73]) shows that CA IV activity—±expression or ±ACZ—does not affect the NBCe1-A current (and therefore transport) induced by identical electrical driving forces [73]. As noted in our discussion of Figure 3, if HCO_3_^−^ or CO_3_^2−^ is the transported ion, little is gained by interconverting HCO_3_^−^ and CO_2_. On the other hand, if CO_2_ is the transported species, this interconversion has major effects on enhancing diffusion.

**Figure 6:**
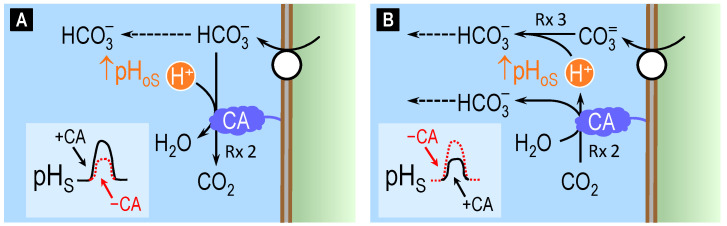
Theory of bicarbonate vs. carbonate efflux across cell membranes. (**A**) The exit of HCO_3_^−^ leads to the consumption of H^+^ (i.e., an increase in pH_oS_; inset, lower left corner: solid black trace) via the CA-catalyzed reaction HCO_3_^−^ + H^+^ → CO_2_ + H_2_O (indicated as ‘Rx 2’) at the outer surface (oS) of the cell membrane. Blocking CA (i.e., applying a CA inhibitor) reduces H^+^ consumption (i.e., the rise in pH_oS_; dashed red trace). (**B**) The exit of CO_3_^2−^ leads to the consumption of H^+^ (i.e., an increase in pH_oS_; solid black trace) via the reaction CO_3_^2−^ + H^+^ → HCO_3_^−^ (indicated as ‘Rx 3’) at the outer surface of the cell membrane. Blocking CA (i.e., applying a CA inhibitor) reduces production of H^+^ (by ‘Rx 2’) for subsequent consumption via ‘Rx 3’. Thus, CA inhibition amplifies the rise in pH_oS_ (dashed red trace). Rx = reaction; the number following the abbreviation ‘Rx’ indicates the numbering of the reaction in the main text. CA = carbonic anhydrase.

Here, in light of our findings that NBCe1-A transports CO_3_^2−^, we suggest that the role of CA IV at the BLM is to minimize local basolateral pH changes caused by CO_3_^2−^ transport. Figure 6 shows the effects of HCO_3_^−^ vs. CO_3_^2−^ efflux on pH_oS_ in the presence (solid black trace) and absence (dashed red trace) of CA IV. If NBCe1-A were to transport HCO_3_^−^, then CA IV would accentuate the alkalinity of the extracellular surface. If NBCe1-A were to transport CO_3_^2−^, CA IV would reduce the alkalinity. In preliminary work, we increased peritubular [K^+^] to depolarize mouse PTs (thereby driving Na^+^ and “HCO_3_^−^” into the PT cell) and observed the expected decrease in pH_oS_. Performing this maneuver in the presence of a peritubular CA inhibitor markedly increased the magnitude of the pH_oS_ decrease. These observations are consistent with the CO_3_^2−^ transport model (Figure 6B). However, because PTs express many acid–base transporters, further investigations—for example, the use of PTs from knockout (KO) mice lacking specific acid–base transporters—are needed.

Regarding a potential physiological role for CA XII at the BLM, we suggest that this enzyme, as CA IV, minimizes pH_oS_ changes at the BLM. 

To summarize the actions of CAs in the PT, the apical CA IV and cytosolic CA II are complementary in accelerating the transmembrane CO_2_ flux [56,57,58]. By converting CO_2_ to the far-more-abundant HCO_3_^−^, CA II also accelerates the flux of “carbon” from the apical to the basolateral membrane. In the process, CA II also provides cytosolic substrates for apical H^+^ extrusion and basolateral “HCO_3_^−^” efflux. The CA IV and XII at the BLM, however, do not play a substantial (or measurable) role in dissipating the “product” of NBCe1-A activity (i.e., the product being the appearance of CO_3_^2−^ in the extracellular nanodomain near NBCe1-A). Instead, these CAs stabilize pH in this nanodomain.

### 6.2. Thick Ascending Limb

**Figure 7:**
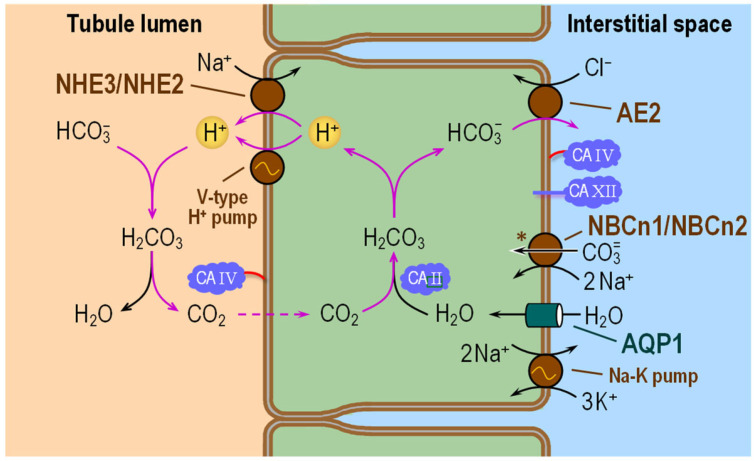
Cell model of HCO_3_^−^ reabsorption in the thick ascending limb. In the lumen, filtered HCO_3_^−^ and H^+^—secreted by NHE3/NHE2 and V-type H^+^ pump—react to form CO_2_ and H_2_O under the catalytic action of CA IV. Apical entry of CO_2_ and basolateral entry of H_2_O via AQP1 lead to the intracellular formation of HCO_3_^−^ and H^+^ under the catalytic action of CA II. AE2 carries HCO_3_^−^ out of the cell across the basolateral membrane. The brown symbol ‘*’ near NBCn1/NBCn2 denotes uncertainty about NBCn1/NBCn2 stoichiometry. For details, see text. NHE3/NHE2 = Na-H exchanger 3/Na-H exchanger 2; V-type H^+^ pump = Vacuolar-type H^+^ pump (or V-ATPase); CA II, CA IV and CA XII = carbonic anhydrase II, IV and XII; AE2 = anion exchanger 2; AQP1 = aquaporin 1; NBCn1/NBCn2 = electroneutral Na/HCO_3_ cotransporter 1/electroneutral Na/HCO_3_ cotransporter 2.

Approximately 10–15% of filtered HCO_3_^−^ is reabsorbed in the TAL. The mechanism of HCO_3_^−^ reabsorption in the TAL (Figure 7) is very similar to that in the S1 and S2 segments of the PT (Figure 5). In the TAL, NHE3/NHE2 and the V-type H^+^ pump extrude H^+^ across the AM [150]. In the lumen, the secreted H^+^ combines with HCO_3_^−^, producing CO_2_ and H_2_O. Because the AM of the TAL is tight to NH_3_ and water and, to date, no CO_2_-conducting membrane proteins (e.g., AQPs) have been identified at the AM, the newly formed CO_2_ enters the cell likely via diffusion only. Apical CA IV and cytosolic CA II, by maximizing the transmembrane CO_2_ gradient across the AM, enhance CO_2_ influx across the AM. Once inside the cytosol, the CO_2_ combines with H_2_O to form HCO_3_^−^ and H^+^. In contrast to PTs, where NBCe1-A exports Na^+^ and CO_3_^2−^ across the BLM, here in the TAL, AE2 exchanges cytosolic HCO_3_^−^ for Cl^−^ at the BLM. In addition to AE2, investigators have identified NBCn1 and (in rat) NBCn2 in the BLM of the TAL [131,151,152,153]. Because NBCn1 and NBCn2 normally operate in an inward direction [154], it seems unlikely that they contribute to HCO_3_^−^ reabsorption per se. Instead, their roles may be to “facilitate” transcellular NH_4_^+^ transport from the TAL to the collecting duct [155,156]. 

The TAL reabsorbs luminal NH_4_^+^ via the Na-K-Cl cotransporter 2 (NKCC2) and the renal outer medullary K^+^ channel (ROMK) [157,158]. Once inside the cell, NH_4_^+^ dissociates into NH_3_ and H^+^. It has been suggested that NBCn1 and NBCn2 neutralize this H^+^, thereby enhancing the formation of intracellular NH_3_ and promoting NH_3_ diffusion through the cell to the BLM. There, the NH_3_ exits the TAL cell, possibly via AQP1 [159], diffuses through the interstitial fluid, and finally crosses the BLM of the α-intercalated cell via RhBG and RhCG [155,156,160]. Consistent with this hypothesis, in vivo and in vitro studies show that metabolic acidosis (MAc) increases NBCn1 expression [131,155,161]. 

Note that in Figure 7 we illustrate NBCn1 and NBCn2 as moving CO_3_^2−^ (rather than HCO_3_^−^) into the cell, consistent with preliminary data from our group. However, because this transporter is electroneutral, in Figure 7 we tentatively illustrate the cotransport of 2 Na^+^ ions. Additional work is needed to elucidate the stoichiometry of NBCn1 and NBCn2.

#### 6.2.1. Subcellular Localizations of CA Isozymes

CA II is present in the cytosol of virtually all renal cells except for those in the tip of Henle’s loop and the thin ascending limb [120]. Within Henle’s loop, CA IV and CA XII are present only in the TAL, with similar localization as in the PT (i.e., CA IV is in both the AM and BLM and CA XII in the BLM.

#### 6.2.2. Functional Interactions with Acid–Base Transporters 

As illustrated in Figure 7, the role of apical CA IV in HCO_3_^−^ reabsorption is to convert luminal HCO_3_^−^ to CO_2_ for uptake across the AM. The role of cytosolic CA II is to promote the consumption of incoming CO_2_, thereby accelerating CO_2_ influx, and to provide cytosolic H^+^ for extrusion by NHE3/NHE2 and the V-type H^+^ pump across the AM, and HCO_3_^−^ for export by AE2 across the BLM. These roles of apical CA IV and cytosolic CA II are similar to those discussed above for the PT.

Some investigators have reported that CA II and AEs can physically interact, thereby stimulating AEs activity [162,163,164,165]. According to other reports, CA IV binds to the 4^th^ extracellular loop of AE1 (probably also AE2 and AE3), also stimulating AE1 activity [166]. However, other studies have shown no evidence of direct binding of CA II to the C-terminus of AE1 [147,167].

The work of Piermarini and colleagues [147] confirmed that liquid-phase AE1-C terminus (Ct) can bind to solid-phase CA II when—as in the earlier work—they fused the AE1-Ct to GST (which, significantly, forms dimers). Piermarini et al. replicated these results for GST-NBCe1-Ct and GST-NDCBE-Ct (collectively SLC4-Ct). However, when they reversed the orientation and applied liquid-phase CA II to solid-phase GST-SLC4-Ct, they observed no binding. Even when CA II was in the solid phase, SLC4-Ct failed to bind in the absence of GST. Thus, the interaction between the Ct of AE1 (and also of NBCe1 and NDCBE) does indeed occur but is not physiologically relevant because it requires that (i) the Ct be fused to GST and (ii) the GST fusion protein be liquid phase.

Moreover, our recent work (Figure 6 and Supplemental Table 3A in ref. [73]) shows that in oocytes expressing AE1 (±CA IV or ±ACZ) and exposed to Cl^−^-free CO_2_/HCO_3_^−^ solution, the presence of CA IV does not affect the rates of pH_i_ recovery [73]. These experiments follow the trans-side rule of Musa-Aziz et al. [56,57,58]: it is legitimate to use pH measurements to assess the impact of a CA on the activity of an acid–base transporter only if the CA and the pH probe are on opposite sides of the membrane (e.g., CA IV on the outside, pH on the inside). The earlier authors added the CA II to the cytosol and measured pH on the same side. In their experiments, they saw that CA II markedly accelerated the AE1-dependent pH_i_ changes. This would have occurred regardless of the effect (if any) of CA II on the transport rate because CA II—interposed between AE1 and the pH_i_ probe—was responsible for translating the HCO_3_^−^ flux into a pH_i_ signal.

Aside from the experimental data, as noted above in our discussion of Figure 3 as well as in the above corresponding section for PT, if HCO_3_^−^ or CO_3_^2−^ is the transported ion, the advantage of a CA-catalyzed interconversion of HCO_3_^−^ and CO_2_ for “HCO_3_^−^“ transport would be small.

Here, in light of our findings that AE1 transports HCO_3_^−^ and that NBCn1 and NBCn2 appear to carry CO_3_^2−^ we propose that the role of CA IV at the BLM is not related to AE2 but to NBCn1 and NBCn2. Thus, we suggest that the CA IV (and possibly CA XII) minimizes pH_oS_ changes at the BLM caused by CO_3_^2−^ entry by NBCn1 and NBCn2 (see insert, lower left corner of panel B of Figure 4: solid black trace). This pH_oS_ stabilization would presumably be beneficial to other nearby pH-sensitive proteins.

### 6.3. Tubules Distal to the Thick Ascending Limb

We have seen that most HCO_3_^−^ reabsorption occurs in the PTs (~80–85%) and the TAL (~10–15%). The remaining ~5–10% of HCO_3_^−^ reabsorption occurs in the distal nephron (i.e., from the macula densa, which marks the beginning of the distal convoluted tubule through the inner medullary collecting duct).

Like the tubule segments from the beginning of the PT to the end of the TAL (which expresses NKCC2), the earliest tubule segment after the macula densa, the distal convoluted tubule 1 (DCT1), has one cell type. This DCT1 cell (which expresses the Na/Cl cotransporter NCC) does not appear to participate in acid–base transport. The next segment, the DCT2, has two cell types, DCT2 cells (with NCC + epithelial Na^+^ channel ENaC) and intercalated cells (ICs), the latter of which do participate in acid–base transport. The third segment, the connecting tubule (CNT), has both CNT cells (ENaC + AQP2) and ICs. The fourth segment, the initial collecting tubule (ICT), also has two cell types, the segment-specific principal cells (PCs; ENaC + AQP2) and ICs. The fifth segment, which begins after the first confluence of ICTs, is identical to the ICT but has a new name, the cortical collecting tubule (CCT) or duct; it has both segment-specific PCs as well as ICs. The sixth post-macula-densa segment is the outer medullary collecting duct (OMCD), which again has segment-specific PCs as well as ICs. Finally, the inner medullary collecting duct (IMCD) has ICs in its initial part, but thereafter IMCD cells (AQP2 + UT-A1) [168,169,170,171,172,173,174]. The proportion of PCs versus ICs varies along the segments of the distal nephron, among and within species, and with physiological conditions [170,172,175].

Among the cell type listed above, only the ICs perform substantial transepithelial acid–base transport, with perhaps some contribution from the IMCD cells. The ICs comprise three subtypes: type-A or α-ICs (dominant in OMCD), type-B or β-ICs (dominant in CNT through CCT), and non-A/non-B [172,175,176].

**Figure 8:**
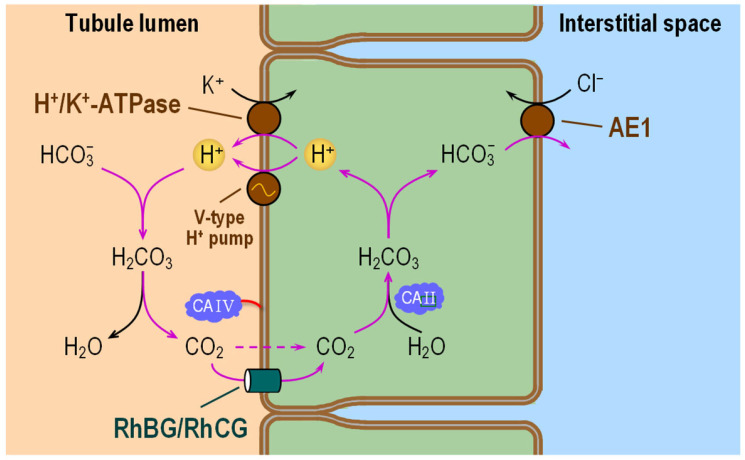
Cell model of HCO_3_^−^ reabsorption in α-intercalated cells of the distal nephron. In the lumen, residual filtered HCO_3_^−^ and H^+^—secreted by H^+^/K^+^-pump and V-type H^+^ pump—react to form CO_2_ and H_2_O under the catalytic action of CA IV. Apical entry of CO_2_ via RhBG/RhCG leads to the intracellular formation of HCO_3_^−^ and H^+^ under the catalytic action of CA II. AE1 carries HCO_3_^−^ out of the cell across the basolateral membrane. V-type H^+^ pump = Vacuolar-type H^+^ pump (or V-ATPase); RhBG/RhCG = Rh family B glycoprotein/Rh family C glycoprotein; CA II and CA IV= carbonic anhydrase II and IV; AE1 = anion exchanger 1.

The α-ICs secrete H^+^ into the tubule lumen and thus are responsible for reabsorbing the remaining 5–10% of filtered HCO_3_^−^ that enters the distal nephron (Figure 8). These cells perform this task by secreting H^+^ into the lumen via the V-type H^+^ pump and the H^+^/K^+^ pump, both on the AM [176,177,178]. CA IV at the AM converts filtered luminal HCO_3_^−^ and secreted H^+^ to CO_2_ and H_2_O. CO_2_ presumably enters the cell via the Rh proteins RhBG and RhCG [160]. In the cytosol, CA II catalyzes the conversion of the incoming CO_2_ and H_2_O to HCO_3_^−^ and H^+^. The newly formed HCO_3_^−^ exits the BLM through the renal variant of AE1 [176,177,178]. Note that the roles of apical CA IV and cytosolic CA II in the α-IC are similar to those discussed above for the PT and TAL cells.

The β-ICs cells secrete HCO_3_^−^ into the tubule lumen [76,179]. Opposite to the α-ICs, the β-ICs express the V-type H^+^ pump in the BLM, and a Cl-HCO_3_ exchanger in the AM. However, this apical Cl-HCO_3_ exchanger is pendrin (SLC26A4).

Non-A/non-B ICs, because they express both the V-type H^+^ pump and pendrin in the AM [169,170], are intermediate between α-ICs and β-ICs (or, some say, a hybrid of the two), perhaps representing an intermediate state of cell differentiation [180]. For example, Purkerson and colleagues observed that MAc causes β-cells to become α-cells [181]. This transformation requires a large extracellular-matrix protein called hensin (from Japanese *henshin*, transformation) or deleted in malignant brain tumors 1 (DMBT1), as well as other proteins [180,182]. Indeed, mice without DMBT1 in the ICs have only a β-IC-like phenotype and develop MAc [183]. Inducing chronic MAc in rats leads to increased number of α-ICs. Similarly, inducing chronic metabolic alkalosis (MAlk) increases the number of β-ICs [180,184,185]. 

A recent study shows that the genetic deletion of NBCe1 (either global or PT-specific KOs) eliminates the typical IC response to MAc (e.g., an increase in α-IC and decrease in β-ICs) and is consistent with the idea of a link between PTs and ICs [186]. 

#### 6.3.1. Subcellular Localizations of CA Isozymes

High amount of CA II is present in the cytosol of all ICs in the distal nephron. CA IV is expressed only in the AM of α-ICs [120,126]. Principal cells also have CA II, but the amount is significantly lower than in the ICs [187].

#### 6.3.2. Functional Interactions with Acid–Base Transporters

The sub-cellular localization of CAs, as well as the presence of a H^+^-secretory mechanism at the AM and a HCO_3_^−^-exit mechanism at the BLM of α-ICs, provide all the tools necessary for these cells to perform H^+^ secretion in conjunction with HCO_3_^−^ reabsorption. Under normal physiological conditions, the contribution of ICs to whole-kidney HCO_3_^−^ reabsorption is relatively small. However, these cells may play a critical role as they “fine tune” urinary acid secretion. Moreover, it appears that these cells can compensate for impaired HCO_3_^−^ reabsorption in the preceding tubules [188]. A relatively low abundance of NCBTs (i.e., known or putative CO_3_^2−^ transporters) on the BLM of ICs correlates with a relatively low expression of membrane-associated CAs at the BLM. This is consistent with our hypothesis that the role of membrane-associated CAs is not so much as to provide substrates to “HCO_3_^−^” transporters as to stabilize pH in the nanodomains near H^+^ and CO_3_^2−^ transporters. It will be informative to re-examine these issues with the accumulation of future data on the expression of NCBTs and CAs in the distal nephron.

## 7. Conclusions

CA activity has long been recognized as critically important for normal physiological function. For this reason, since the discovery of the first CA, investigators have focused considerable attention on the identification of CAs in a wide range of cells, how these CAs respond to physiological and pathophysiological challenges, and—using tools of molecular biophysics—the mechanisms of action of the CAs. Because of the fundamental role of CAs in acid–base homeostasis, some of these studies have focused on the interactions of CAs with acid–base transporters. As illustrated for the PT, TAL, and α-IC, the role of apical CA IV and cytosolic CA II is to accelerate CO_2_ influx across the AM, to speed “carbon” diffusion from AM to BLM, and to provide H^+^ and HCO_3_^−^ as substrates for apical H^+^ extrusion and basolateral “HCO_3_^−^” transport via members of the SLC4 family. Whereas the role of apical CA IV is to accelerate CO_2_ influx across apical membranes (i.e., PT, TAL, and α-IC), the role of basolateral CA IV in the renal PT is to provide the H^+^ necessary to titrate the newly transported CO_3_^2−^ to HCO_3_^−^. By preventing an excessive rise in pH_oS_, basolateral CA IV (and CA XII) protects pH-sensitive processes near NBCe1-A at the outer surface of the BLM. We suggest that it is likely that membrane-associated CAs play similar buffering roles when coupled with other members of the SLC4 that carry CO_3_^2−^. These membrane-associated CAs would similarly minimize pH_oS_ changes near the H^+^-extrusion mechanism but not near HCO_3_^−^ transporters (where they would accentuate pH_oS_ changes).

In the case of α- and β-ICs, the interconversion from one subtype to the other must involve substantial changes in the expression of CA IV, changes that coordinate with those in the acid–base transporters.

## Figures and Tables

**Table 1:** Classification of human SLC4 family members that carry “HCO_3_^−^” in the kidney. mRNA and protein expression levels were classified based on the study [96]. Very low: ≤5 nTPM; low: 5 ≤ 15 nTPM; medium: 15 ≤ 50 nTPM; high: >50 nTPM. * N/A means either not tested or not detected. nTPM = normalized tags per million.

Gene (Protein) Name	mRNA Expression				Protein Expression			
	Very Low	Low	Medium	High	Low	Medium	High	N/A *
*SLC4A1* (AE1)				x		x		
*SLC4A2* (AE2)			x			x		
*SLC4A3* (AE3)		x						x
*SLC4A4* (NBCe1)				x			x	
*SLC4A5* (NBCe2)	x					x		
*SLC4A7* (NBCn1)		x					x	
*SLC4A8* (NDCBE)	x							x
*SLC4A9* (AE4)			x					x
*SLC4A10* (NBCn2)	x						x	

**Table 2:** Classification of human α-CA family members in the kidney. mRNA and protein expression levels were classified based on the study [96]. Very low: ≤1 nTPM; low: 1 ≤ 15 nTPM; medium: 15 ≤ 50 nTPM; high: >50 nTPM. * N/A means either not tested or not detected. nTPM = normalized tags per million. Note that no data are available for *CA15* because this gene has not been identified in humans [97].

Gene (Protein) Name	mRNA Expression				Protein Expression			
	Very Low	Low	Medium	High	Low	Medium	High	N/A *
*CA1* (CA I)		x						x
*CA2* (CA II)				x			x	
*CA3* (CA III)		x						x
*CA4* (CA IV)			x		x			
*CA5A* (CA VA)	x							x
*CA5B* (CA VB)		x				x		
*CA6* (CA VI)	x							x
*CA7* (CA VII)	x							x
*CA8* (CA VIII)		x						x
*CA9* (CA IX)	x							x
*CA10* (CA X)		x						x
*CA11* (CA XI)		x						x
*CA12* (CA XII)				x			x	
*CA13* (CA XIII)		x				x		
*CA14* (CA XIV)	x							x

## Data Availability

Not applicable.
